# Milt androgen profile and evaluation of sperm morpho-functional characteristics of wild-caught and farmed European eels (*Anguilla anguilla*)

**DOI:** 10.1007/s10695-025-01494-y

**Published:** 2025-04-07

**Authors:** Laura Gentile, Bálint Lóránt Hausz, Antonio Casalini, Nadia Govoni, Pietro Emmanuele, Albamaria Parmeggiani, Domenico Ventrella, Maria Laura Bacci, Oliviero Mordenti, Alberto Elmi

**Affiliations:** 1 Department of Veterinary Medical Sciences, Alma Mater Studiorum - University of Bologna, Via Tolara Di Sopra 50, 40064 Ozzano Dell’Emilia, Italy; 2 Department of Veterinary Sciences, University of Pisa, Viale Delle Piagge 2, 56124 Pisa, Italy

**Keywords:** Eel, 11-ketotestosterone, Testosterone, Milt, Spermatozoa, Male fertility

## Abstract

Evaluating milt samples before their preservation and/or reproductive use is critical to correctly develop assisted reproductive strategies for teleost species. This is especially true in case of “critically endangered” species, like the European eel (*Anguilla anguilla*), whose life cycle is not closed yet, leading to the necessity of capturing younglings from natural populations for both research and productive purposes. Despite androgens being involved in spermatogenesis and playing pivotal roles in the pre-migratory morphological and physiological changes, their quantification in different biological tissues is still to be refined in European eels. The aim of this study was to evaluate the feasibility of extraction and quantification of testosterone (T) and 11-ketotestosterone (11-KT) from male European eel milts. The secondary aim was to investigate potential differences in androgens’ profiles and/or functional characteristics of spermatozoa between wild-caught and farmed specimens. Twenty-four male European eels obtained from the North Adriatic region (14 wild-caught and 10 farmed) underwent a maturation-inducing hormonal treatment for milt production; blood and milt samples were then collected for androgens quantification upon ELISA and RIA methodologies. Spermatozoa were also evaluated for viability and objective motility. Plasma showed average T and 11-KT levels of 2.32 and 6.88 ng/mL, respectively, while milt 0.24 (T) and 1.09 ng/mL (11-KT). Significantly higher spermatozoa concentrations and motility were observed in farmed eels’ milts. This work contributes to deepening the knowledge on teleost physiology, being the first report quantifying T and 11-KT from European eels’ seminal plasma and comparing the quality of milts of farmed vs wild specimens.

**Supplementary Information:**

The online version contains supplementary material available at 10.1007/s10695-025-01494-y.

## Introduction

Aquaculture is one of the fastest agricultural growing businesses (FAO 2022), with farmed fish accounting for nearly 50% of the global fish consumption. The European eel, *Anguilla anguilla*, is a species with high potential market in aquaculture (Lovatelli and Holthus 2008), but the population has declined by 98% between 1980 and 2010, leading to its “critically endangered” status according to the International Union for Conservation of Nature (IUCN) Red List (Pike et al. 2018; Richards et al. 2020). Generally speaking, the aquaculture sector thrives when it comes to species whose entire life cycle can be fully reproduced in captivity (Olesen et al. 2003); unfortunately, this is currently not true for European eels, since sourcing of juvenile fish for farming purposes is still based on capturing wild specimens. These animals will eventually undergo hormonal-induced maturation in order to produce milt (Mordenti et al. 2018; Palstra et al. 2022). In such scenario, developing hatching and larval weaning technologies to complete the captive life cycle and enable production to meet markets demands would be crucial, trying to alleviate pressure from the natural population. Eels are a catadromous species, and because of their complex life cycle and peculiarities, they evolutionarily developed very complex hormonal control mechanisms. At the end of their continental phase, eels undergo a pubertal event (Aroua et al. 2006), comprising of morphological and physiological changes referred to as silvering (Haro 2003), in preparation to a long reproductive migration and their oceanic phase. In order to be able to mimic these conditions in captivity and induce spermatogenesis, hormonal treatments are necessary (Tomkiewicz et al. 2011; Mordenti et al. 2018). Different protocols have been developed, with the most common requiring weekly injections of hCG, ensuring a longer period of sperm production and effective synchronization with the maturation of female oocytes. This leads to a spawning season that can extend over 2–3 months (Pérez et al. 2000; Tomkiewicz et al. 2011; Mordenti et al. 2018). Alongside the weekly injections, hCG is also administered before sperm retrieval, also known as “primer” or “booster” injection, to ensure high-quality mature spermatozoa (Di Biase et al. 2017).

Before using the collected milt, it is important to evaluate both quantitative and qualitative aspects of the spermatozoa for a successful egg fertilization and for high quality larvae production. The latter is particularly important when developing assisted reproductive techniques for species that rarely spawn naturally in captivity. Among a high number of factors impacting the reproductive success, like housing settings (temperature, pH, dO_2_, salinity) and the number and quality of oocytes, the evaluation of milts before each reproductive event may help ruling out functional alterations, better predicting the reproductive outcomes (Gallego and Asturiano 2019). The quality of eel sperm has already been investigated and documented by different studies (Locatello et al. 2018; Gallego and Asturiano 2019). The advent of new technologies like computer-assisted sperm analysis (CASA), which automates sperm quality assessment, has permitted the estimation of a high number of sperm kinetic parameters, which have been linked to fertilization and hatching success in different fish species (Gallego et al. 2013; Elmi et al. 2023). The analysis of steroid hormone profiles is often done to test animals’ maturity, since spermatogenesis is a complex developmental process in which androgens play critical roles (Miura and Miura 2003). The biosynthesis of androgens from cholesterol is catalyzed by several steroidogenic enzymes under the control of pituitary gonadotropins (Yaron et al. 2003; Levavi-Sivan et al. 2010). In male fish, 11-ketotestosterone (11-KT) has been established as a unique and potent androgen pivotal for sex determination, secondary sexual characteristics development, and spermatogenesis (Borg 1994). It is well-known that in teleost species, the production of androgens and plasma levels increase with the progression of maturity status (Miura et al. 1991). The spermatogonial mitotic proliferation before the meiotic event is promoted by 11-KT (Miura and Miura 2003), with the latter being important also for the control of the silvering-related changes in Anguillid species (Rohr et al. 2001). On the other hand, the renewal of spermatogonial stem cells is controlled by Estradiol- 17β (E- 17β) (Miura et al. 1999); during its biosynthesis, the aromatase (cyp19a1a/b) is a key enzyme, converting testosterone (*T*) into estradiol (Parmeggiani et al. 2015; Tokarz et al. 2015). Therefore, while T is not the most characteristic androgen in teleost species, its presence is necessary for spermatogenesis as a precursor of the physiologically active 11-KT and E- 17β, and in fact, 17‐methyltestosterone is actively used to induce maturation of female eels (Mordenti et al. 2018).

The most frequently used biological sample to quantify steroid hormone profiles is blood, which requires an invasive sampling technique. However, a growing interest for the search of alternative matrices obtained using less invasive techniques has been noted lately, based on the principle that steroid hormones can be found in different biological samples. As a matter of fact, literature suggests the possibility to assess animals welfare and sexual maturity using a wide array of biological samples both in mammals (Ventrella et al. 2020; Elmi et al. 2020; Aniballi et al. 2023) and in teleost (Sebire et al. 2007). Using seminal plasma for the assessment of welfare and sexual hormones may present a good solution in the field of alternative and opportunistic samplings, as its sampling in necessary for artificial insemination and is performed by simple stripping technique from spermiating males (Butts et al. 2014; Di Biase et al. 2016; Koumpiadis et al. 2021). Of course, a strong correlation between seminal and plasmatic hormone levels would be necessary to propose seminal plasma as a valid alternative to blood.

Therefore, the aim of this study is to evaluate the feasibility of extraction and quantification of steroid hormones from male European eel milts, and to preliminary verify correlations between T and 11-KT levels in blood plasma and seminal plasma. Secondary aims are to investigate potential correlations of androgen levels with the functional characteristics of the spermatozoa, and to compare both androgen profiles and spermatozoa quality between wild-caught and farmed eels from the Nord-Italian regions.

## Materials and methods

### Ethical approval

All fish were kept and handled following the guidelines for experimental procedures in accordance with the European Union regulations concerning the protection of experimental animals (DIR 2010/63/UE). This study was approved by the Ethics Committee of Bologna University (ID 1157).

### Fish handling

A total of 24 males (*N* = 24) were used for this study: 10 (*n* = 10) were caught in a lagoon near Vene di Bellocchio (Emilia Romagna, Italy) during the autumn–winter migration period (November–February), with body weight of 135.9 ± 9.2 g and length of 43.9 ± 0.8 cm; 14 (*n* = 14) were collected from a farm (Colombo Fish Farm, Milan, Italy), in the same period, with 140.8 ± 11.5 g of body weight and 45.8 ± 1.4 cm of body length.

All eels were moved to the facilities of the Department of Veterinary Medical Sciences (Cesenatico, Italy), where they were tagged using fish-tags (FLOY TAG Mod Floy T-Bar Anchor). Animals were maintained in two separate 700 L tanks with natural sea water, equipped with separate recirculation systems, thermostats, and coolers, and covered to maintain constant darkness. Eels were gradually acclimated during the first 7 days to reach the standard experimental conditions: temperature of 15 ± 0.5 °C, salinity of 32‰, pH 7.8, and dO_2_ of 9 ppm (Mordenti et al. 2014).

### Hormonal induction

After the acclimation period, in order to induce milt production, animals were anaesthetized with phenoxyethanol (400 ppm) and weighed before being treated with 1.0 IU/g of hCG (Corulon, 5000 UI, Intervet, Segrate, Milan, Italy), once a week, using previously published protocol (Mordenti et al. 2013).

### Blood and milt collection

Samples were only collected once for each animal after reaching sexual maturation, confirmed by the production of milt volumes extractable by stripping. The duration of the hCG treatment was recorded and included in the following analyses.

Blood (0.5 mL) was collected into EDTA test tubes from the posterior cardinal vein, using a 1 mL sterile syringe equipped with a 26 G needle, upon anaesthesia. Samples were transferred within 2 h to the physiology laboratories of the Department of Veterinary Medical Sciences in Ozzano dell’Emilia (BO), at 4 °C, where they were centrifuged at 2000 × *g* for 15 min; plasma was collected and stored at − 20 °C until analysis.

Milt collection was performed at the same time, 24 h after the last administration of hCG. Before the collection, the urogenital area was accurately cleaned with deionized water and wiped dry. Two mL of milt was obtained by delicate pressure on the abdomen and collected in sterile microtubes; the first drops were not collected to avoid urine and feces contamination (Sørensen et al. 2013). A 400 µL aliquot of each ejaculate was immediately diluted 1:10 with a dedicated non-activating storage medium (P1 medium) (Asturiano et al. 2004; Peñaranda et al. 2010a) for the functional evaluations; the remaining volume was kept undiluted for endocrinologic assays. Regardless, all samples were transferred to the physiology laboratories at 4 °C. Once in the lab, the undiluted samples were centrifuged at 7500 × *g* for 5 min to collect the seminal plasma, which was stored at − 20 °C for steroid quantifications. The 1:10 diluted aliquots were processed as follows.

### Spermatozoa evaluation

Upon arrival, the 1:10 diluted aliquots were evaluated for sperm concentration and viability.

Viability was assessed using the Eosin-Nigrosin staining method (Elmi et al. 2017): 10 µL of staining solution were added to 10 µL of each sample, and 8 µL were immediately smeared on a glass microscope slide for analysis. The percentage of live cells (undyed spermatozoa/all spermatozoa) was evaluated on a minimum of 400 spermatozoa.

For the assessment of sperm concentration, each aliquot was further diluted 1:100 or 1:1000 in P1 medium, and a Thoma haemocytometer was used with × 40 magnification and contrast phase microscopy (Nikon Eclipse E600; Nikon Corporation, Tokyo, Japan). Spermatozoa were counted in five larger squares (0.2 × 0.2 × 0.1 mm) of the haemocytometer (Elmi et al. 2023). Results were reported as spermatozoa × 10^9^/mL.

Sperm objective motility was assessed using a CASA system (Hamilton Thorne CEROS II; Animal Motility II, Software Version 1.9, Beverly, MA, USA) with dedicated settings for fish samples; analyses were performed as previously reported (Elmi et al. 2023). For this evaluation, 200 µL of the diluted samples were further diluted 1:5 with the activating extender. The latter, as well as all plastic lab ware, Leja 4 chamber slides (IMV Technologies, L’Aigle France), and the removable CASA stage were kept at 4 °C for at least 1 h prior analyses. A minimum of 500 spermatozoa were captured with a frame capture speed of 60 Hz and a total frame count of 45. The analyzed parameters were total motility (tMot, %), curvilinear velocity (VCL, µm/s), straight-line velocity (VSL, µm/s), average path velocity (VAP, µm/s), distance average path (DAP, µm), distance curved line (DCL, µm), distance straight line (DSL, µm), percentage of linearity (LIN, %), percentage of straightness (STR, %) Wobble coefficient (WOB, %), mean amplitude of lateral head displacement (ALH, µm), and beat cross frequency (BCF, Hz) (Gallego et al. 2013).

### Quantification of androgens

#### 11-ketotestosterone

11-KT concentration was determined by competitive enzyme immunoassay (Cayman Chemical EIA kit, Ann Arbor, MI, USA) according to the manufacturer’s instructions. Extraction of steroids from blood plasma (5 µL) and seminal plasma (50 µL) was performed with ethyl acetate/hexane (50:50 v/v) in 4 X the sample volume, using pyrex glass tubes according to the method recommended by the producer. The samples were vortexed twice for 10 s and frozen to allow the separation of layers. The upper layer was transferred to a clean glass tube and left to evaporate under an air-stream suction hood. The extracts were then dissolved in the kit buffer (5 mL), and 50 µL was used for the analysis in triplicates. The plate was read at 412 nm using Infinite F50 reader (Tecan—Grodig, Austria). A series of dilutions (1:1–1:8) were made for both blood and seminal samples, which were found to be parallel to the standard assay curve. Cross reactions of other steroids with antiserum raised against 11-KT were 11-KT (100%), Adrenosterone (2.9%), 4-Androsten- 11β, 17β-diol- 3-one (0.01%), and 5α-Androstan- 17β-ol- 3-one, 5α-Androsten- 3β, 17β-diol, Testosterone (< 0.01%). The detection limit of assay was 1.43 pg/mL; the intra-assay coefficient of variation was 5.8%. The results were expressed as ng/mL of blood or milt plasma.

#### Testosterone

Testosterone concentrations were measured in blood plasma and seminal plasma by radioimmunoassay (Parmeggiani et al. 2015). Briefly, 100 µL of blood plasma and 600 µL of seminal plasma were extracted with diethyl ether (approximately 1:10 v/v) in Pyrex glass tubes and mixed for 30 min on a rotary mixer. The glass tubes were centrifuged at 2000 g for 15 min, and the supernatants evaporated until dryness under an air-stream suction hood. The dry extracts were reconstituted in the assay buffer (PBS, 0.1% BSA, pH 7.4) for a final measurement of T (related to 200 µL seminal plasma equivalent and 15 µL blood plasma equivalent). Tritiated T (0.1 mL, 30 pg/tube; 83.4 Ci/mmol; PerkinElmer, USA) was added, followed by rabbit anti-testosterone serum (0.1 mL, 1:50,000 produced in our laboratory). After incubation overnight, antibody-bound and -unbound steroids were separated by charcoal–dextran solution (charcoal 0.25%, dextran 0.02% in phosphate buffer), and the tubes were centrifuged for 15 min at 3000 × g. All the supernatant was decanted, and the radioactivity was immediately measured using a *β*-scintillation counter (Packard C1600, Perkin Elmer, USA).

Cross reactions of various steroids with antiserum raised against testosterone were T (100%), dihydrotestosterone (25.44%), androstenedione (0.6%), progesterone and cortisol (< 0.0001%). The sensitivity of the assay was 3.5 pg/tube, and the intra-assay coefficient of variation was 6.7%. In order to determine the parallelism between hormone standards and endogenous hormone in eel seminal plasma or blood plasma, casually selected samples containing high concentrations was serially diluted (1:1–1:8) with the assay buffer. A regression analysis was used to determine parallelism between the two hormone levels in the same assay. The results were expressed as ng/mL of blood or milt plasma.

### Statistical analysis

Statistical analyses and graphical representations were performed using GraphPad Prism 9.0 (GraphPad Software Inc., San Diego, CA, USA). The descriptive results are reported as mean and standard deviations (SD). Shapiro–Wilk test was applied to verify normality of distribution. For the differential analysis of steroid hormone profiles and spermatozoa morpho-functional characteristics, data were grouped by source of the animals (wild vs farmed), and Mann–Whitney *U* tests were applied. Correlations were investigated using the Spearman rank test for non-parametrical data. All statistical analyses were performed with fixed significance level of *p* < 0.05.

## Results

The results of the descriptive analysis for 11-KT, T, and milt morpho-functional parameters, as well as the spermatozoa kinematic parameters, are reported in the supplementary material (Tables S1 and S2, respectively). Testosterone was not quantifiable in 5 milt samples and in 1 blood plasma sample (Table S1). These cases have been excluded from the correlation analysis for the specific missing parameter, respectively. On the other hand, 11-KT was successfully quantified in all samples.

**Fig. 1:**
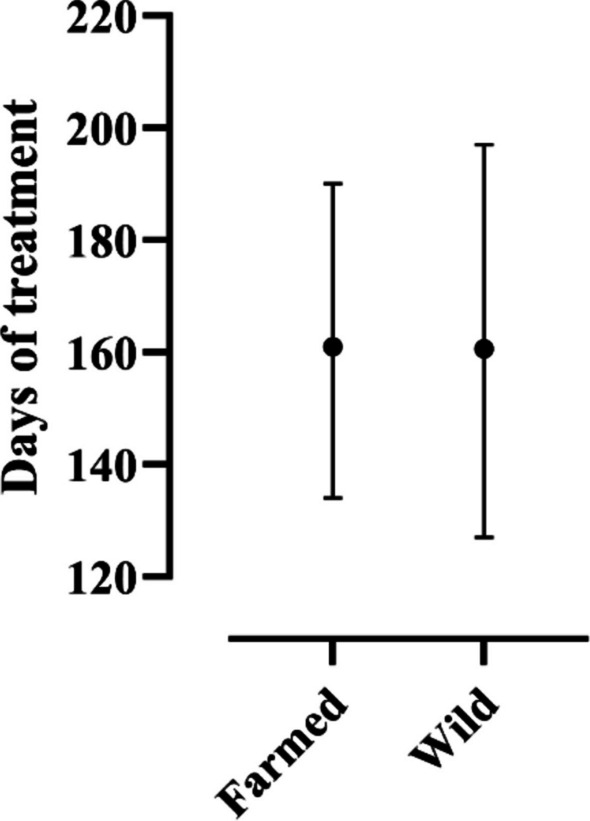
Duration of the hCG treatment for animals upon origin categorization. Dots indicate the mean, error bars min–max values

The duration of the hCG treatment (Fig. 1) was, in average, 161 days (range 134–190) for farmed eels (*n* = 14) and 160.6 days (range 127–197) for the wild-caught ones (*n* = 10).

### Evaluation of steroid concentrations

Testosterone was quantifiable in 96% of plasma samples (23/24) and 79% of milt ones (19/24).

**Fig. 2:**
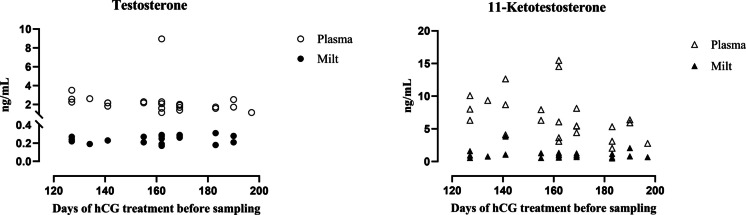
Distribution of testosterone (T) and 11-ketotestosterone (11 KT) concentrations in the blood and milt of European eels, in relation to the duration of the of hCG treatment (considering the range of 120–200 days)

For both T and 11-KT, a nearly ten-fold difference was observed between plasma and milt levels, with milt showing lower androgens concentrations (Fig. 2). While the concentration of both androgens in blood demonstrates an overall decreasing trend in relation to the days of hCG treatment before sampling, they remain constant in the milt.

**Fig. 3:**
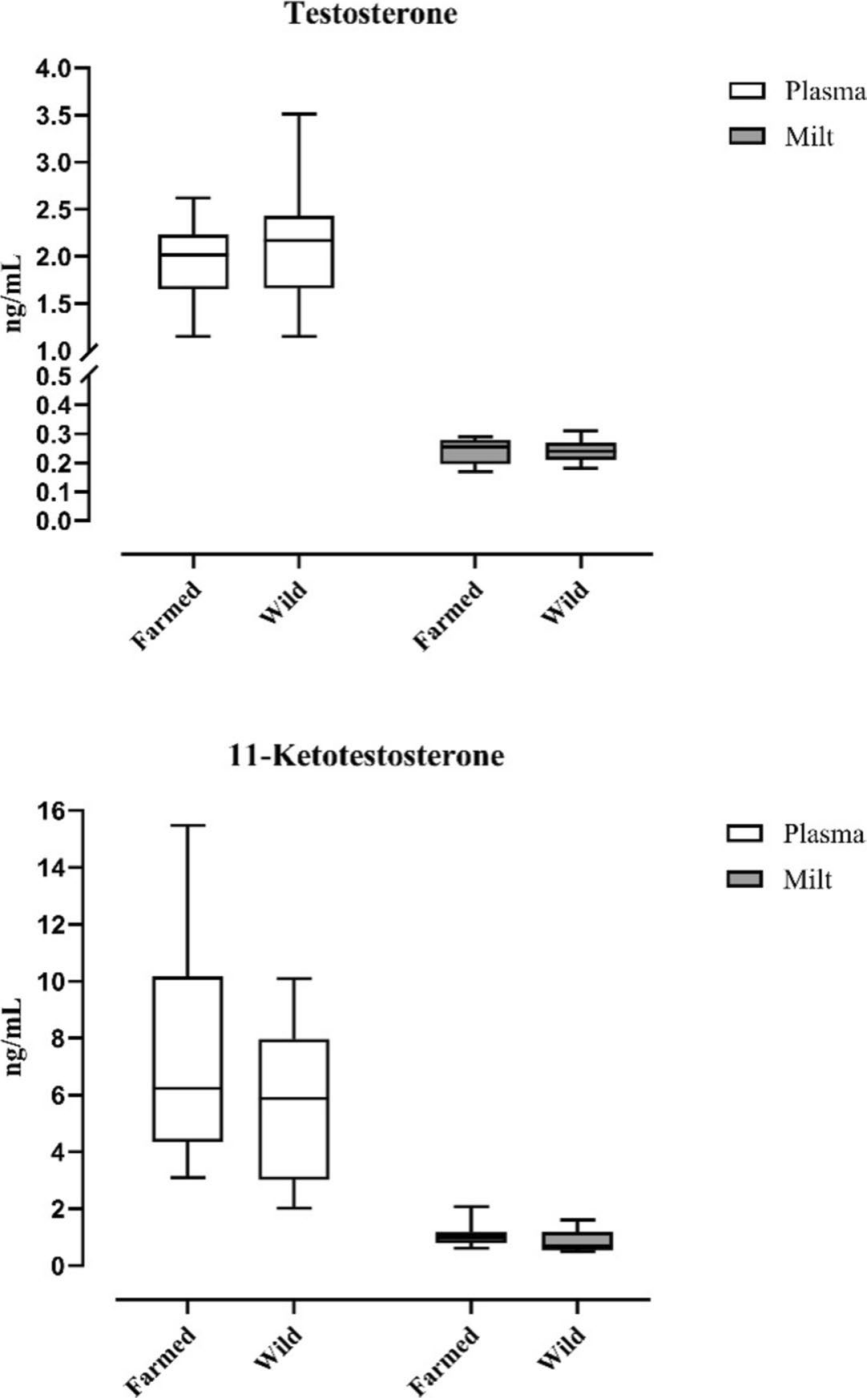
Graphical representations of the concentrations of testosterone and 11-ketotestosterone in blood plasma and milt of farmed and wild European eels. Concentrations are expressed in nanograms per milliliter (ng/mL). Differences between groups (farmed vs. wild) were assessed using the Mann–Whitney *U* test, with a statistical significance level set at *p* < 0.05

Blood plasma averagely contained 6.88 ng/mL of 11-KT, with a higher mean in farmed eels (7.70 ng/mL) than in wild ones (5.73 ng/mL), yet not statistically relevant (Fig. 3). No statistical differences were also observed for 11-KT in plasma and in milt between the two groups (plasma: *p* = 0.2847; milt: *p* = 0.1257). Average T level in plasma was 2.32 ng/mL, while 0.24 ng/mL in milt (Fig. 3). No significant differences between farmed and wild eels were recorded (plasma: *p* = 0.7692; milt: *p* = 0.8535).

### Evaluation of morpho-functional characteristics of the spermatozoa based on origin

**Fig. 4:**
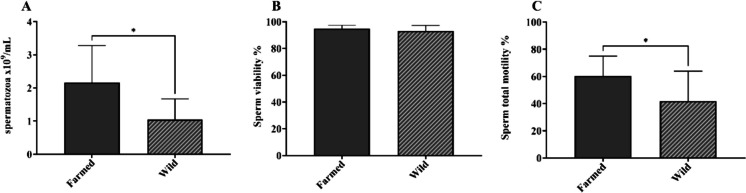
Graphical representation of sperm concentration (**A**), viability (**B**), and total motility (**C**) upon comparison between farmed and wild eels. The asterisk (*) indicates statistically significant differences between groups (farmed vs. wild) at the level of *p* < 0.05. The kinematic parameters of sperm motility are reported in Table S2

All milt samples included in the study were successfully analyzed for morpho-functional sperm parameters. A mean concentration of 1.70 × 10^9^ spermatozoa/mL was observed, with farmed eels showing significantly higher values when compared to wild ones (*p* = 0.0135, Fig. 4A). Overall mean viability was 94.10%, without statistically significant differences between farmed and wild eels (*p* = 0.3946, Fig. 4B). Farmed eels showed a significantly higher mean percentage of motile spermatozoa (*p* = 0.0294, Fig. 4C).

### Correlation analysis

**Fig. 5:**
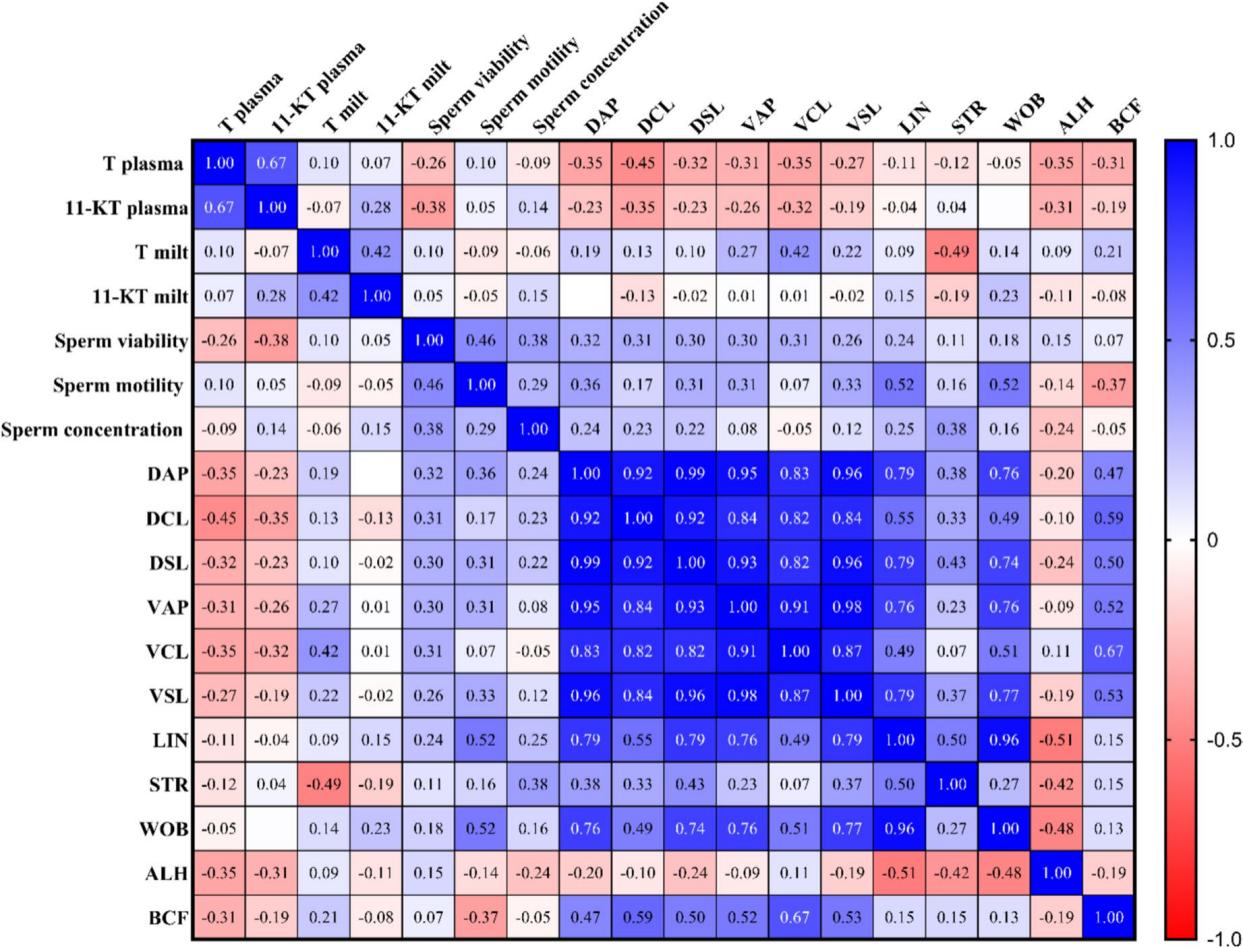
Results of the correlation analysis in terms of *ρ* coefficients of the Spearman rank test. Data are reported all together, without categorization per origin of eels. DAP, distance average path; DCL, distance curved line; DSL, distance straight line; VAP, average path velocity; VCL, curvilinear velocity; VSL, straight-line velocity; LIN, percentage of linearity; STR, percentage of straightness; WOB, Wobble coefficient; ALH, mean amplitude of lateral head displacement; BCF, beat cross frequency

Correlation analysis was conducted both by dividing the data based on the origin of the animals and without division. The former did not reveal any difference of the parameters correlated between farmed and wild-caught eels, thus only the results of the Spearman rank test on undivided-data are shown (Fig. 5); the corresponding *p* values are included in the supplementary material (Table S4).

No significant correlations were detected between blood plasma and milt androgen concentrations (T *ρ* = 0.1042, *p* = 0.6807; 11-KT *ρ* = 0.2802, *p* = 0.1848). A correlation was observed between the two steroids in plasma (*ρ* = 0.6734; *p* = 0.0004), but not in milt (*ρ* = − 0.0660, *p* = 0.0715). Regarding the functional parameters of the spermatozoa, a positive correlation was found between sperm motility and viability (*ρ* = 0.4585, *p* = 0.0242). Sperm motility also positively correlated with LIN (*ρ* = 0.5220, *p* = 0.0089) and WOB (*ρ* = 0.5212, *p* = 0.0090). Interestingly, a negative correlation was observed between plasma T and DCL (*ρ* = − 0.4509, *p* = 0.0308), but no other significant correlations were found between androgens in the different biological samples and the spermatic parameters (concentration, motility, viability). A great number of kinematic parameters were correlated to each other due to the algorithmic structure of the CASA evaluation program.

## Discussion

To the best of the authors’ knowledge, the present work reports the first successful quantification of T and 11-KT in the seminal plasma of the European eel. The chosen extraction protocol was initially developed and used on mammal specimens (Gaiani et al. 1984) then modified and applied to teleost species (Parmeggiani et al. 2015). Locatello et al. (2018) used a similar protocol to extract T from blood plasma, and the same procedure was recently used for steroid extraction from mammalian seminal plasma (Aniballi et al. 2023). The present study demonstrates the applicability of the protocol for extraction and quantification of steroids from the milt of male European eels.

Looking at the study design, it is important to state that animals were only sampled once, after reaching iatrogenic hormonal-induced sexual maturation, using a protocol well documented by literature. This implies differences across animals in the number of days of hormonal treatments prior sampling. As the objective of the study was not to test the changes in androgen levels with multiple timepoints, each animal represented one biological replica, and the differences in days of treatment accounted for the natural physiological variability of milt production and sperm formation. Nonetheless, upon division of animals based on their origin, the mean duration of the treatment prior sampling was identical (161 days), allowing for a safe comparison.

The average quantity of testosterone found in blood plasma is in accordance with the findings of other studies. Sato et al. (2006) and Sudo et al. (2012) reported similar values for Japanese eels (*Anguilla japonica*). The latter study reports similar values to the present one not only for T but also for plasmatic 11-KT. Similar T values in blood are reported for European eels also in the works of Peyon and colleagues (1997) and Peñaranda and colleagues (2016). Due to the lack of more bibliography quantifying 11-KT in the milt of anguillid species, further comparison of the results is not possible.

Initial high concentrations of T in the plasma are then used by catalyzing enzymes to produce 11-KT and other steroids including estrogens. Existing literature reports higher T concentrations in blood before milt production extractable by stripping, after which a decreasing trend is observable as T is transformed into 11-KT and E- 17β (Baynes and Scott 1985; Peñaranda et al. 2016). In the present study, 11-KT quantity significantly correlated to T in blood, and the 11-KT:T ratio reflects an advanced stage of milt production and sperm formation at the time of sampling. This can possibly explain T quantities in the milt samples below the threshold of RIA sensibility. The observed decreasing trend for both androgens in blood plasma is also in accordance with previous findings of another research group (Peñaranda et al. 2016). Despite the current experimental design not being opted for the analysis in function of time, sampling eels after different number of hCG treatment days revealed that the decreasing trend of T and 11-KT in the blood plasma does not affect the androgen concentrations in the milt, which remain nearly constant. This finding is reasonable, considering the role of steroid hormones in endogenous signaling along the reproductive tract and in exogenous signaling as pheromones (Garner and Hafez 2000; Stacey 2003; Huertas et al. 2006). No significant correlation was present between androgen concentrations in the milt, probably because of difference in the controlling mechanism regulating the release of T and 11-KT from the testis into the milt itself. In mammals, steroid dependency of the myoid and Sertoli cells is met by the production of interstitial Leydig cells, which also release steroids into seminiferous tubules. FSH stimulates the secretion of androgen-binding-proteins in the Sertoli cells, which carry androgens into the epididymis for the highly androgen-dependent epithelial cells (Garner and Hafez 2000). In teleost species, the milt also acts as an exogenous pheromone, thus its androgen content is regulated by multiple factors, which presumably also obscures the correlation between the two analyzed fluids.

For the profound investigation of androgen correlations between the blood and milt, a more focused study with larger sampling pool and synchronized hormonal induction with multiple sampling time-points would be necessary, leading to a better profiling of androgen concentrations and its dynamics in the milt. The latter is now possible also for European eels without the use of more invasive techniques requiring samples from the testis. In any case, a potential correlation between steroid levels in milt and plasma remains an open question, which could potentially lead to the development of steroid hormonal profiling protocols causing significantly lower stress for the animal and facilitate maturation determination for teleost species used in aquaculture.

In the present study, high spermatic viability was observed both for farmed and wild eels without statistically significant differences between the two origins. Also, this finding can be explained by the advanced stages of spermatogenesis prior to spermiation, as higher spermatic viability (> 75%) is reported to be associated to the later stages of spermatogenesis (Peñaranda et al. 2010a, b). Farmed eels showed significantly higher concentration of spermatozoa and spermatic motility (Fig. 2). Eels are capable of de novo lipid synthesis even during prolonged starvation (Abraham et al. 1984; Gnoni and Muci 1990), but the enzymatic activity involved is dependent on diet’s fatty acid composition (Butts et al. 2015). Polyunsaturated fatty acids (PUFA), especially eicosapentaenoic acid [EPA; C20:5(n − 3)], are reported to be synthetized in the liver from the precursor metabolites, to be transferred into the testis and used for cell sperm cell membranes (Baeza et al. 2015), with a positive correlation between EPA percentages and best motility (Butts et al. 2015). In a previous study comparing the functional characteristics of European eel’s spermatozoa based on the origin of the animal before hormonal treatment, the authors reported greater spermatocrit values and sperm longevity in wild caught eels (Locatello et al. 2018). The result of this study shows an overall greater sperm quality in eels obtained from farms. The difference in the results might be contributed to the number of participating animals, differences in the study design, or the application of automated motility analysis in the present study. A potential explanation to the greater sperm quality in eels obtained from farms might be attributed to the diets consumed prior to participating in the study and the trophic conditions. It is possible that the higher concentration of spermatozoa and greater motility of farmed eels is due to better overall trophic conditions in farms, as the amount of food given to the eels is optimized for the growth of the animals (Cabrita et al. 2014). Hence, in latter stages of the life cycle when eels stop feeding, these animals are in a better nutritive status for the biosynthesis of the specific lipids necessary for the different stages of testis development and sperm/milt formation (Baeza et al. 2015; Butts et al. 2015). Although the wild males used for this test were captured in an area known to have good trophic conditions (Mordenti et al. 2014, 2023; Emmanuele et al. 2020; Gentile et al. 2022), we cannot assume that these specimens spent their entire continental phase there; thus, we cannot be sure of the type of feeding they were exposed to. Therefore, from an aquacultural perspective, it appears that male eels raised on farms can demonstrate better sperm quality, which makes them better candidates for artificial breeding programs.

Other studies reported that while 11-KT increases milt volume, it does not correlate with spermatozoa concentration nor spermatic motility in spotted wolffish (*Anarhichas minor*) (Kugathaj 2005) or rainbow trout (*Oncorhynchus mykiss*) (Koldras et al. 1996). Accordingly, the spermatic motility positively correlated to the spermatozoa viability, but no statistically significant correlation was found between androgens in the two analyzed fluids and the spermatozoa concentration, viability, or motility, also in the case of European eel.

## Conclusions

In conclusion, the present study suggests how the proposed extraction and analytical protocols allowed for the quantification, for the first time to the best of the author’s knowledge, of testosterone and 11-ketotestosterone in seminal plasma of male European eels with different origins (wild/farmed) undertaking maturation-inducing hCG treatment. Upon activation, eels obtained from farms showed significantly higher spermatozoa concentration and motility than eels caught from the wild, most probably due to differences in the trophic conditions between the natural habitat and the diets used in aquaculture farms.

## Supplementary Information

Below is the link to the electronic supplementary material.

Supplementary file 1 (DOCX 36.7 KB)  

## Data Availability

All data generated in the present study are available from the corresponding author upon reasonable request.

## References

[CR1] Abraham S, Hansen HJ, Hansen FN (1984) The effect of prolonged fasting on total lipid synthesis and enzyme activities in the liver of the European eel (*Anguilla anguilla*). Comp Biochem Physiol B 79:285–289. 10.1016/0305-0491(84)90027-0 6150805 10.1016/0305-0491(84)90027-0

[CR2] Aniballi C, Elmi A, Govoni N et al (2023) Influence of age and seasonality on boar seminal plasma steroids quantification: a preliminary study. Vet World 2150–2157. 10.14202/vetworld.2023.2150-2157 10.14202/vetworld.2023.2150-2157 PMC10668552 38023267

[CR3] Aroua S, Schmitz M, Baloche S et al (2006) Endocrine evidence that silvering, a secondary metamorphosis in the eel, is a pubertal rather than a metamorphic event. Neuroendocrinology 82:221–232. 10.1159/000092642 10.1159/000092642 16679776

[CR4] Asturiano JF, Pérez L, Garzón DL et al (2004) Physio-chemical characteristics of seminal plasma and development of media and methods for the cryopreservation of European eel sperm. Fish Physiol Biochem 30:283–293. 10.1007/s10695-005-1553-x

[CR5] Baeza R, Mazzeo I, Vílchez MC et al (2015) Relationship between sperm quality parameters and the fatty acid composition of the muscle, liver and testis of European eel. Comp Biochem Physiol A Mol Integr Physiol 181:79–86. 10.1016/j.cbpa.2014.11.022 25483240 10.1016/j.cbpa.2014.11.022

[CR6] Baynes SM, Scott AP (1985) Seasonal variations in parameters of milt production and in plasma concentration of sex steroids of male rainbow trout (*Salmo gairdneri*). Gen Comp Endocrinol 57:150–160. 10.1016/0016-6480(85)90211-4 3972242 10.1016/0016-6480(85)90211-4

[CR7] Borg B (1994) Androgens in teleost fishes. Comp Biochem Physiol C Pharmacol Toxicol Endocrinol 109:219–245. 10.1016/0742-8413(94)00063-G

[CR8] Butts IAE, Baeza R, Støttrup JG et al (2015) Impact of dietary fatty acids on muscle composition, liver lipids, milt composition and sperm performance in European eel. Comp Biochem Physiol A Mol Integr Physiol 183:87–96. 10.1016/j.cbpa.2015.01.015 25638567 10.1016/j.cbpa.2015.01.015

[CR9] Butts IAE, Sørensen SR, Politis SN et al (2014) Standardization of fertilization protocols for the European eel, *Anguilla anguilla*. Aquaculture 426–427:9–13. 10.1016/j.aquaculture.2014.01.020

[CR10] Cabrita E, Martínez-Páramo S, Gavaia PJ et al (2014) Factors enhancing fish sperm quality and emerging tools for sperm analysis. Aquaculture 432:389–401. 10.1016/j.aquaculture.2014.04.034

[CR11] Di Biase A, Casalini A, Emmanuele P et al (2016) Controlled reproduction in *Anguilla anguilla* (L.): comparison between spontaneous spawning and stripping-insemination approaches. Aquac Res 47:3052–3060. 10.1111/are.12755

[CR12] Di Biase A, Lokman PM, Govoni N et al (2017) Co-treatment with androgens during artificial induction of maturation in female eel, *Anguilla anguilla*: effects on egg production and early development. Aquaculture 479:508–515. 10.1016/j.aquaculture.2017.06.030

[CR13] Elmi A, Ventrella D, Barone F et al (2017) Thymbra capitata (L.) Cav. and Rosmarinus officinalis (L.) essential oils: in vitro effects and toxicity on swine spermatozoa. Molecules 22:2162. 10.3390/molecules22122162 29211030 10.3390/molecules22122162 PMC6149686

[CR14] Elmi A, Galligioni V, Govoni N et al (2020) Quantification of hair corticosterone, DHEA and testosterone as a potential tool for welfare assessment in male laboratory mice. Animals 10:2408. 10.3390/ani10122408 33339323 10.3390/ani10122408 PMC7766173

[CR15] Elmi A, Casalini A, Bertocchi M et al (2023) Comparative evaluation of the effects of different activating media and temperatures on European eel (*Anguilla anguilla*) sperm motility assessed by computer assisted sperm analysis. Res Vet Sci 164:105045. 10.1016/j.rvsc.2023.105045 37812988 10.1016/j.rvsc.2023.105045

[CR16] Emmanuele P, Casalini A, Pisati D et al (2020) Artificial reproduction of *Anguilla anguilla*: evaluation of biometrics characteristics of a population from Valle Campo Lagoon, Comacchio (Italy). Aquac Int 28:777–790. 10.1007/s10499-019-00494-z

[CR17] FAO (2022) The state of world fisheries and aquaculture 2022: towards blue transformation. FAO, Rome. 10.4060/cc0461en

[CR18] Gaiani R, Chiesa F, Mattioli M et al (1984) Androstenedione and testosterone concentrations in plasma and milk of the cow throughout pregnancy. Reproduction 70:55–59. 10.1530/jrf.0.0700055 10.1530/jrf.0.0700055 6694152

[CR19] Gallego V, Asturiano JF (2019) Fish sperm motility assessment as a tool for aquaculture research: a historical approach. Rev Aquac 11:697–724. 10.1111/raq.12253

[CR20] Gallego V, Carneiro PCF, Mazzeo I et al (2013) Standardization of European eel (*Anguilla anguilla*) sperm motility evaluation by CASA software. Theriogenology 79:1034–1040. 10.1016/j.theriogenology.2013.01.019 23465287 10.1016/j.theriogenology.2013.01.019

[CR21] Garner DL, Hafez ES (2000) Spermatozoa and seminal plasma. In: Reproduction in farm animals. John Wiley & Sons, Ltd, pp 96–109. 10.1002/9781119265306.ch7

[CR22] Gentile L, Casalini A, Emmanuele P et al (2022) Gonadal development in European eel populations of North Adriatic lagoons at different silvering stages. Appl Sci 12:2820. 10.3390/app12062820

[CR23] Gnoni GV, Muci MR (1990) De novo fatty acid synthesis in eel-liver cytosol. Comp Biochem Physiol Part B Comp Biochem 95:153–158. 10.1016/0305-0491(90)90263-S

[CR24] Haro A (2003) Downstream migration of silver-phase *Anguillid* eels. In: Aida K, Tsukamoto K, Yamauchi K (eds) Eel Biology. Springer Japan, Tokyo, pp 215–222

[CR25] Huertas M, Scott AP, Hubbard PC et al (2006) Sexually mature European eels (*Anguilla anguilla* L.) stimulate gonadal development of neighbouring males: possible involvement of chemical communication. Gen Comp Endocrinol 147:304–313. 10.1016/j.ygcen.2006.01.017 16545383 10.1016/j.ygcen.2006.01.017

[CR26] Koldras M, Loir M, Maisse G, Gac FL (1996) Study of the composition of seminal fluid and of sperm motility along the genital tract, during a spawning season, in the rainbow trout (*Oncorhynchus mykiss*). Aquat Living Resour 9:337–345. 10.1051/alr:1996036

[CR27] Koumpiadis P, Sganga DE, Politis SN et al (2021) Sperm production and quality in European eel (*Anguilla anguilla*) in relation to hormonal treatment. Reprod Domest Anim 56:1497–1505. 10.1111/rda.14011 34478180 10.1111/rda.14011

[CR28] Kugathaj S (2005) Effect of 11-ketotestosterone on sperm production by male spotted wolffish (*Anarhichas minor*). Master thesis, Universitetet i Tromsø

[CR29] Levavi-Sivan B, Bogerd J, Mañanós EL et al (2010) Perspectives on fish gonadotropins and their receptors. Gen Comp Endocrinol 165:412–437. 10.1016/j.ygcen.2009.07.019 19686749 10.1016/j.ygcen.2009.07.019

[CR30] Locatello L, Bertotto D, Cerri R et al (2018) Sperm quality in wild-caught and farmed males of the European eel (*Anguilla anguilla*). Anim Reprod Sci 198:167–176. 10.1016/j.anireprosci.2018.09.016 30301621 10.1016/j.anireprosci.2018.09.016

[CR31] Lovatelli A, Holthus PF (2008) Capture-based aquaculture: global overview. FAO, Rome

[CR32] Miura T, Miura CI (2003) Molecular control mechanisms of fish spermatogenesis. Fish Physiol Biochem 28:181–186. 10.1023/B:FISH.0000030522.71779.47

[CR33] Miura T, Yamauchi K, Takahashi H, Nagahama Y (1991) Hormonal induction of all stages of spermatogenesis in vitro in the male Japanese eel (*Anguilla japonica*). Proc Natl Acad Sci 88:5774–5778. 10.1073/pnas.88.13.5774 2062857 10.1073/pnas.88.13.5774 PMC51960

[CR34] Miura T, Miura C, Ohta T et al (1999) Estradiol-17β stimulates the renewal of spermatogonial stem cells in males. Biochem Biophys Res Commun 264:230–234. 10.1006/bbrc.1999.1494 10527870 10.1006/bbrc.1999.1494

[CR35] Mordenti O, Biase AD, Bastone G et al (2013) Controlled reproduction in the wild European eel (*Anguilla anguilla*): two populations compared. Aquac Int 21:1045–1063. 10.1007/s10499-012-9611-8

[CR36] Mordenti O, Casalini A, Mandelli M, Di Biase A (2014) A closed recirculating aquaculture system for artificial seed production of the European eel (*Anguilla anguilla*): technology development for spontaneous spawning and eggs incubation. Aquac Eng 58:88–94. 10.1016/j.aquaeng.2013.12.002

[CR37] Mordenti O, Emmanuele P, Casalini A et al (2018) Effect of aromatable androgen (17-methyltestosterone) on induced maturation of silver European eels (*Anguilla anguilla*): oocyte performance and synchronization. Aquac Res 49:442–448. 10.1111/are.13475

[CR38] Mordenti O, Gentile L, Emmanuele P et al (2023) Evaluation of the reproductive performance of females of *Anguilla anguilla* characterized by different levels of silvering. Appl Sci 13:10718. 10.3390/app131910718

[CR39] Olesen I, Gjedrem T, Bentsen HB et al (2003) Breeding programs for sustainable aquaculture. J Appl Aquac 13:179–204. 10.1300/J028v13n03_01

[CR40] Palstra AP, Jéhannet PJ, Heinsbroek LT, Swinkels W (2022) Five years of optimizing the assisted reproduction protocol for European eel: what worked and what didn’t? In: Proceedings of 12th World Congress on Genetics Applied to Livestock Production (WCGALP). Wageningen Academic Publishers, pp 2028–2030. 10.3920/978-90-8686-940-4_488

[CR41] Parmeggiani A, Govoni N, Zannoni A et al (2015) Effect of photoperiod on endocrine profiles and vitellogenin expression in European eels *Anguilla anguilla* during artificially induced ovarian development. Theriogenology 83:478–484. 10.1016/j.theriogenology.2014.10.008 25459031 10.1016/j.theriogenology.2014.10.008

[CR42] Peñaranda DS, Pérez L, Gallego V et al (2010) European eel sperm diluent for short-term storage. Reprod Domest Anim Zuchthyg 45:407–415. 10.1111/j.1439-0531.2008.01206.x 10.1111/j.1439-0531.2008.01206.x 18954399

[CR43] Peñaranda DS, Pérez L, Gallego V et al (2010) Molecular and physiological study of the artificial maturation process in European eel males: from brain to testis. Gen Comp Endocrinol 166:160–171. 10.1016/j.ygcen.2009.08.006 19699741 10.1016/j.ygcen.2009.08.006

[CR44] Peñaranda DS, Morini M, Tveiten H et al (2016) Temperature modulates testis steroidogenesis in European eel. Comp Biochem Physiol A Mol Integr Physiol 197:58–67. 10.1016/j.cbpa.2016.03.012 27013359 10.1016/j.cbpa.2016.03.012

[CR45] Pérez L, Aturiano JF, Tomás A et al (2000) Induction of maturation and spermiation in the male European eel: assessment of sperm quality throughout treatment. J Fish Biol 57:1488–1504. 10.1111/j.1095-8649.2000.tb02227.x

[CR46] Peyon P, Baloche S, Burzawa-Gérard E (1997) Investigation into the possible role of androgens in the induction of hepatic vitellogenesis in the European eel:in vivo andin vitro studies. Fish Physiol Biochem 16:107–118. 10.1007/BF00004668

[CR47] Pike C, Vicki C, Gollock M (2018) IUCN Red list of threatened species: *Anguilla anguilla*. 10.2305/IUCN.UK.2020-2.RLTS.T60344A152845178.en

[CR48] Richards JL, Sheng V, Yi CW et al (2020) Prevalence of critically endangered European eel (Anguilla anguilla) in Hong Kong supermarkets. Sci Adv 6:eaay0317. 10.1126/sciadv.aay0317 32181342 10.1126/sciadv.aay0317 PMC7056311

[CR49] Rohr DH, Lokman PM, Davie PS, Young G (2001) 11-ketotestosterone induces silvering-related changes in immature female short-finned eels, *Anguilla australis*. Comp Biochem Physiol A Mol Integr Physiol 130:701–714. 10.1016/S1095-6433(01)00402-0 11691606 10.1016/s1095-6433(01)00402-0

[CR50] Sato N, Kawazoe I, Suzuki Y, Aida K (2006) Effects of temperature on vitellogenesis in Japanese eel *Anguilla japonica*. Fish Sci 72:961–966. 10.1111/j.1444-2906.2006.01244.x

[CR51] Sebire M, Katsiadaki I, Scott AP (2007) Non-invasive measurement of 11-ketotestosterone, cortisol and androstenedione in male three-spined stickleback (*Gasterosteus aculeatus*). Gen Comp Endocrinol 152:30–38. 10.1016/j.ygcen.2007.02.009 17412342 10.1016/j.ygcen.2007.02.009

[CR52] Sørensen S, Gallego V, Pérez L et al (2013) Evaluation of methods to determine sperm density for the European eel, *Anguilla anguilla*. Reprod Domest Anim 48:936–944. 10.1111/rda.12189 23772654 10.1111/rda.12189

[CR53] Stacey N (2003) Hormones, pheromones and reproductive behavior. Fish Physiol Biochem 28:229–235. 10.1023/B:FISH.0000030540.99732.2c

[CR54] Sudo R, Tosaka R, Ijiri S et al (2012) 11-ketotestosterone synchronously induces oocyte development and silvering-related changes in the Japanese eel, *Anguilla japonica*. Zoolog Sci 29:254–259. 10.2108/zsj.29.254 22468835 10.2108/zsj.29.254

[CR55] Tokarz J, Möller G, Hrabě de Angelis M, Adamski J (2015) Steroids in teleost fishes: a functional point of view. Steroids 103:123–144. 10.1016/j.steroids.2015.06.011 26102270 10.1016/j.steroids.2015.06.011

[CR56] Tomkiewicz J, Kofoed TMN, Pedersen JS (2011) Assessment of testis development during induced spermatogenesis in the European eel *Anguilla anguilla*. Mar Coast Fish 3:106–118. 10.1080/19425120.2011.556902

[CR57] Ventrella D, Elmi A, Bertocchi M et al (2020) Progesterone and cortisol levels in blood and hair of wild pregnant red deer (*Cervus Elaphus*) hinds. Animals 10:143. 10.3390/ani10010143 31963117 10.3390/ani10010143 PMC7022734

[CR58] Yaron Z, Gur G, Melamed P et al (2003) Regulation of fish gonadotropins. In: International review of cytology. Academic Press, pp 131–185. 10.1016/S0074-7696(05)25004-0 10.1016/s0074-7696(05)25004-0 12696592

