# Supplementary material for: Milt androgen profile and evaluation of sperm morpho-functional characteristics of wild-caught and farmed European eels (Anguilla anguilla)
Source: Fish Physiol Biochem. 2025 Apr 7;51(2):78. doi: 10.1007/s10695-025-01494-y (PMC11976806; doi:10.1007/s10695-025-01494-y)
Supplement: Supplementary file 1 — (DOCX 36.7 KB) [file 10695_2025_1494_MOESM1_ESM.docx]

SUPPLEMENTARY MATERIAL

**Milt androgen profile and evaluation of sperm morpho-functional characteristics of wild caught and farmed European eels (*Anguilla anguilla*)**

Laura Gentile ^1,§^, Bálint Lóránt Hausz ^1,§^, Antonio Casalini ^1^, Nadia Govoni ^1^, Pietro Emmanuele ^1^, Albamaria Parmeggiani ^1^, Domenico Ventrella ^1,^*, Maria Laura Bacci ^1^, Oliviero Mordenti ^1^, Alberto Elmi ^1,2^

^1^Department of Veterinary Medical Sciences, Alma Mater Studiorum - University of Bologna, Via Tolara di Sopra 50, 40064 Ozzano dell’Emilia, Italy

^2^Department of Veterinary Sciences, University of Pisa, Viale delle Piagge 2, 56124 Pisa, Italy

^§^ Authors equally contributed

*Corresponding author: domenico.ventrella2@unibo.it

Table S1. Descriptive analysis of the sperm morpho-functional characteristics and hormone concentrations in plasma and milt.

|  | Wild | | | | | Farmed | | | | |
| --- | --- | --- | --- | --- | --- | --- | --- | --- | --- | --- |
|  | n | Min | Max | Mean | SD | n | Min | Max | Mean | SD |
| Sperm Viability (%) | 10 | 84.25 | 98.25 | 93.05 | 4.02 | 14 | 90.50 | 98.25 | 94.86 | 2.44 |
| Sperm Total Motility (%) | 10 | 6.10 | 72.30 | 41.91 | 20.89 | 14 | 30.10 | 81.00 | 60.51 | 13.96 |
| Sperm concentration (×10^9^/mL) | 10 | 0.21 | 1.79 | 1.05 | 0.59 | 14 | 0.54 | 4.83 | 2.16 | 1.08 |
| T plasma (ng/mL) | 9 | 1.15 | 3.51 | 2.13 | 0.63 | 14 | 1.15 | 8.95 | 2.45 | 1.85 |
| T milt (ng/mL) | 7 | 0.18 | 0.31 | 0.24 | 0.04 | 12 | 0.17 | 0.29 | 0.24 | 0.04 |
| 11-KT plasma (ng/mL) | 10 | 2.03 | 10.09 | 5.73 | 2.44 | 14 | 3.09 | 15.47 | 7.70 | 3.87 |
| 11-KT milt (ng/mL) | 10 | 0.49 | 1.61 | 0.86 | 0.36 | 14 | 0.62 | 3.85 | 1.25 | 0.80 |

T= testosterone; 11-KT= 11-Ketotestosterone

Table S2. Descriptive statistics of the kinematic parameters as recorded by CASA.

|  | Wild | | | | Farmed | | | |
| --- | --- | --- | --- | --- | --- | --- | --- | --- |
| Kinematic parameter | Min | Max | Mean | SD | Min | Max | Mean | SD |
| ALH (μm) | 5.2510 | 6.2984 | 5.6115 | 0.3537 | 5.3093 | 6.2420 | 5.5591 | 0.2279 |
| BCF (Hz) | 26.1114 | 37.0720 | 32.6087 | 3.1324 | 27.6742 | 37.6512 | 30.5407 | 2.4559 |
| DAP (μm) | 49.6475 | 76.3938 | 65.4263 | 7.2411 | 58.1317 | 82.7476 | 68.3895 | 6.8614 |
| DCL (μm) | 87.5196 | 111.3531 | 99.3491 | 7.0052 | 90.1592 | 116.4528 | 100.4460 | 7.1022 |
| DSL (μm) | 44.4792 | 70.1515 | 58.8851 | 6.8006 | 48.9733 | 75.0268 | 61.1974 | 6.7405 |
| LIN (%) | 49.9887 | 62.1994 | 58.4982 | 3.4974 | 54.1986 | 64.9202 | 60.3568 | 3.3602 |
| STR (%) | 83.9322 | 90.5648 | 88.4604 | 1.7599 | 84.5205 | 91.3197 | 88.4962 | 1.8011 |
| VAP (μm/s) | 75.5989 | 118.1837 | 102.4867 | 11.8216 | 88.5915 | 129.6427 | 105.7240 | 11.1366 |
| VCL (μm/s) | 132.5651 | 175.4313 | 155.6721 | 12.3884 | 139.4266 | 181.9518 | 154.7397 | 11.0826 |
| VSL (μm/s) | 67.5808 | 107.9127 | 92.2342 | 11.1004 | 79.6521 | 118.2814 | 94.9615 | 10.6167 |
| WOB (%) | 56.4897 | 68.7466 | 65.6220 | 3.5258 | 62.2651 | 72.0729 | 67.6961 | 3.1677 |

ALH: mean amplitude of lateral head displacement; BCF: beat cross frequency; DAP: distance average path; DCL: distance curved line; DSL: distance straight line; LIN: percentage of linearity; STR: percentage of straightness; VAP: average path velocity; VCL: curvilinear velocity; VSL: straight-line velocity; WOB: Wobble coefficient.

| Animal | Origin | Beginning treatment | Date of sampling | Days of treatment |
| --- | --- | --- | --- | --- |
| 9V | Farmed | 2020.01.13 | 2020.06.30 | 169 |
| 6V | Farmed | 2019.12.16 | 2020.06.23 | 190 |
| 5V | Farmed | 2020.01.13 | 2020.06.30 | 169 |
| 4V | Farmed | 2020.02.10 | 2020.06.23 | 134 |
| 33V | Farmed | 2019.12.16 | 2020.06.23 | 190 |
| 32V | Farmed | 2020.02.10 | 2020.06.30 | 141 |
| 30V | Farmed | 2020.01.13 | 2020.06.23 | 162 |
| 2V | Farmed | 2020.01.13 | 2020.06.23 | 162 |
| 28V | Farmed | 2020.01.13 | 2020.06.23 | 162 |
| 27V | Farmed | 2020.01.13 | 2020.06.30 | 169 |
| 26V | Farmed | 2020.02.10 | 2020.06.30 | 141 |
| 24V | Farmed | 2020.02.10 | 2020.06.30 | 141 |
| 23V | Farmed | 2020.01.13 | 2020.06.23 | 162 |
| 16V | Farmed | 2020.01.13 | 2020.06.23 | 162 |
| 32R | Wild | 2020.01.13 | 2020.06.16 | 155 |
| 29R | Wild | 2020.02.10 | 2020.06.16 | 127 |
| 28R | Wild | 2020.02.10 | 2020.06.16 | 127 |
| 27R | Wild | 2019.12.16 | 2020.06.30 | 197 |
| 26R | Wild | 2020.01.13 | 2020.06.30 | 169 |
| 25R | Wild | 2019.12.16 | 2020.06.16 | 183 |
| 24R | Wild | 2020.01.13 | 2020.06.16 | 155 |
| 23R | Wild | 2019.12.16 | 2020.06.16 | 183 |
| 22R | Wild | 2020.02.10 | 2020.06.16 | 127 |
| 21R | Wild | 2019.12.16 | 2020.06.16 | 183 |

Table S3. List of animals with their origin and duration of hCG treatment.

Table S4. *p* values of the Spearman rank test correlating all evaluated parameters.

|  | T plasma | 11-KT plasma | T milt | 11-KT milt | Sperm viability | Sperm motility | Sperm concentration | DAP | DCL | DSL | VAP | VCL | VSL | LIN | STR | WOB | ALH | BCF |
| --- | --- | --- | --- | --- | --- | --- | --- | --- | --- | --- | --- | --- | --- | --- | --- | --- | --- | --- |
| T plasma |  |  |  |  |  |  |  |  |  |  |  |  |  |  |  |  |  |  |
| 11-KT plasma | *0.000 |  |  |  |  |  |  |  |  |  |  |  |  |  |  |  |  |  |
| T milt | 0.681 | 0.788 |  |  |  |  |  |  |  |  |  |  |  |  |  |  |  |  |
| 11-KT milt | 0.756 | 0.185 | 0.072 |  |  |  |  |  |  |  |  |  |  |  |  |  |  |  |
| Sperm viability | 0.236 | 0.068 | 0.686 | 0.825 |  |  |  |  |  |  |  |  |  |  |  |  |  |  |
| Sperm motility | 0.637 | 0.810 | 0.703 | 0.814 | *0.024 |  |  |  |  |  |  |  |  |  |  |  |  |  |
| Sperm concentration | 0.681 | 0.506 | 0.809 | 0.496 | 0.070 | 0.176 |  |  |  |  |  |  |  |  |  |  |  |  |
| DAP | 0.097 | 0.271 | 0.433 | 0.989 | 0.131 | 0.082 | 0.268 |  |  |  |  |  |  |  |  |  |  |  |
| DCL | *0.031 | 0.089 | 0.592 | 0.531 | 0.134 | 0.432 | 0.290 | *0.000 |  |  |  |  |  |  |  |  |  |  |
| DSL | 0.136 | 0.281 | 0.681 | 0.915 | 0.149 | 0.134 | 0.293 | *0.000 | *0.000 |  |  |  |  |  |  |  |  |  |
| VAP | 0.152 | 0.226 | 0.255 | 0.973 | 0.151 | 0.137 | 0.698 | *0.000 | *0.000 | *0.000 |  |  |  |  |  |  |  |  |
| VCL | 0.104 | 0.132 | 0.076 | 0.981 | 0.142 | 0.750 | 0.827 | *0.000 | *0.000 | *0.000 | *0.000 |  |  |  |  |  |  |  |
| VSL | 0.207 | 0.382 | 0.367 | 0.915 | 0.221 | 0.120 | 0.565 | *0.000 | *0.000 | *0.000 | *0.000 | *0.000 |  |  |  |  |  |  |
| LIN | 0.619 | 0.845 | 0.727 | 0.471 | 0.261 | *0.009 | 0.243 | *0.000 | *0.005 | *0.000 | *0.000 | *0.015 | *0.000 |  |  |  |  |  |
| STR | 0.589 | 0.846 | *0.034 | 0.366 | 0.605 | 0.449 | 0.065 | 0.070 | 0.111 | *0.034 | 0.273 | 0.734 | 0.077 | *0.013 |  |  |  |  |
| WOB | 0.804 | 0.982 | 0.579 | 0.275 | 0.406 | *0.009 | 0.453 | *0.000 | *0.014 | *0.000 | *0.000 | *0.011 | *0.000 | *0.000 | 0.205 |  |  |  |
| ALH | 0.101 | 0.143 | 0.716 | 0.617 | 0.486 | 0.520 | 0.262 | 0.353 | 0.653 | 0.260 | 0.684 | 0.619 | 0.385 | *0.010 | *0.043 | *0.019 |  |  |
| BCF | 0.146 | 0.368 | 0.388 | 0.712 | 0.753 | 0.072 | 0.823 | *0.020 | *0.002 | *0.012 | *0.009 | *0.000 | *0.008 | 0.494 | 0.477 | 0.538 | 0.363 |  |

T: testosterone; 11-KT: 11-ketotestosterone; DAP: distance average path; DCL: distance curved line; DSL: distance straight line; VAP: average path velocity; VCL: curvilinear velocity; VSL: straight-line velocity; LIN: percentage of linearity; STR: percentage of straightness; WOB: Wobble coefficient; ALH: mean amplitude of lateral head displacement; BCF: beat cross frequency.
